# Impact of selenium addition to the cadmium-zinc-telluride matrix for producing high energy resolution X-and gamma-ray detectors

**DOI:** 10.1038/s41598-021-89795-z

**Published:** 2021-05-14

**Authors:** Utpal N. Roy, Giuseppe S. Camarda, Yonggang Cui, Ge Yang, Ralph B. James

**Affiliations:** 1 Brookhaven National Laboratory, Upton, NY 11973 USA; 2 Savannah River National Laboratory, Aiken, SC 29808 USA; 3 North Carolina State University, Raleigh, NC 27695-7909 USA; 4 Present Address: Savannah River National Laboratory, Aiken, SC 29808 USA

**Keywords:** Materials science, Physics

## Abstract

Both material quality and detector performance have been steadily improving over the past few years for the leading room temperature radiation detector material cadmium-zinc-telluride (CdZnTe). However, although tremendous progress being made, CdZnTe still suffers from high concentrations of performance-limiting defects, such as Te inclusions, networks of sub-grain boundaries and compositional inhomogeneity due to the higher segregation coefficient of Zn. Adding as low as 2% (atomic) Se into CdZnTe matrix was found to successfully mitigate many performance-limiting defects and provide improved compositional homogeneity. Here we report record-high performance of Virtual Frisch Grid (VFG) detector fabricated from as-grown Cd_0.9_Zn_0.1_Te_0.98_Se_0.02_ ingot grown by the Traveling Heater Method (THM). Benefiting from superior material quality, we achieved superb energy resolution of 0.77% at 662 keV (as-measured without charge-loss correction algorithms) registered at room temperature. The absence of residual thermal stress in the detector was revealed from white beam X-ray topographic images, which was also confirmed by Infra-Red (IR) transmission imaging under cross polarizers. Furthermore, neither sub-grain boundaries nor their networks were observed from the X-ray topographic image. However, large concentrations of extrinsic impurities were revealed in as-grown materials, suggesting a high likelihood for further reduction in the energy resolution after improved purification of the starting material.

## Introduction

Nuclear radiation detectors, especially those for x- and gamma-rays, have multi-purpose technological applications ranging from homeland security, non-proliferation, astrophysics and medical imaging^1–6^ and are becoming critically important for a diverse range of additional applications. To fulfill the needs, the quest for cost-effective high performing radiation detector materials has continued since the 1970s. The scarcity of finding new materials for such applications is due to the stringent requirements for the material properties. An ideal material for radiation detector applications should be composed of elements with high atomic number to ensure adequate absorption of ionizing radiation. For semiconductor detector materials operating at room temperature, high voltage is needed in order to increase the charge collection efficiency. As a consequence, the bandgap of candidate detector materials should be adequately large to fulfill the requirement of high resistivity (> 10^10^ Ω-cm) at room temperature to achieve low dark current without cooling. In addition to the large bandgap, the material should also have excellent charge-transport characteristics to ensure full charge collection. Owing to the relatively high penetration depth for high energy gamma rays as compared to conventional low energy photon detectors, thick detectors are necessary to ensure sufficient absorption of incident photons. Hence, the material should possess low concentrations of defects to maximize the flow of radiation-induced charge carriers through the detector volume to the respective electrodes. After intense research for over three decades, only a handful of materials have evolved satisfying the requisites for room temperature radiation detector applications. HgI_2_, CdTe, Cd_0.9_Zn_0.1_Te (CZT) and TlBr^7–13^ are among the materials which attracted the most attention. In recent years, halide perovskites have gained interest for radiation detector applications^14,15^. Among others, CsPbBr_3_ is perhaps the most promising perovskite material and an energy resolution of 3.8 (± 0.2) % at 662 keV was reported for a 1.24-mm thick detector^14^. However, ionic migration under applied bias^15^ might hamper the long-term stability of functional CsPbBr_3_ detectors.

Although a lot of progress has been made to search for alternative room temperature radiation detector materials, CZT is still being widely recognized as the gold standard for this type of application mainly due to its high energy resolution performance at room temperature. However, CZT still heavily suffers from compositional inhomogeneity due to its non-unity segregation coefficient of Zn in the CdTe matrix^16^. Meanwhile, CdTe and CZT possess very high concentrations (> 10^5^ cm^−3^) of Te inclusions in the CdTe/CZT matrix^17,18^, and these inclusions are known to have an adverse effect on the detector performance, especially for thick detectors^18^. Despite these disadvantages, CZT is still dominating the commercial domain for the past two and a half decades. The other major drawback with CZT is the sub-grain boundary network. The sub-grain boundaries are generally individual dislocations arranged in planes, called dislocation walls, and are heavily distributed in the bulk of the CZT matrix^18,19^. It is known that the sub-grain boundaries can be formed in CZT ingots due to the thermal stress during crystal growth and subsequent cooling process^19^. In addition, these sub-grain boundaries are usually decorated with deleterious defects such as Te inclusions and impurities^18,19^. Consequently, these sub-grain boundary networks act as charge trapping centers, severely deteriorating the charge transport properties and causing spatial inhomogeneity in the charge transport. To this end, the spatial inhomogeneity of charge transport properties, due to high concentration of Te inclusions and sub-grain boundaries, causes broadening of the photo peak and can severely degrade the detector performance^18^. As a result, both the energy resolution and detection efficiency of the devices are compromised due to the presence of these defects for the present-day CZT materials. It is extremely difficult to eliminate completely these defects because of the poor thermo-physical properties of CZT. However, the sizes and concentrations of Te inclusions can be significantly reduced by post-growth annealing, which has become a routine process to improve the quality of CZT. It should be noted that the annealing process potentially create a new type of star-like defect, commonly known as punching defects. These start-like defects are 50–100 times larger in diameter than that of typical Te inclusions. They are invisible in IR transmission microscopy and can severely hinder the charge transport^20^. All these defects are responsible for spatial inhomogeneity of charge transport properties that eventually degrades the detector performance^21,22^. For example, the spatial variation of the mobility-life time product for electrons [(µτ)_e_] over 0.8 × 0.8 mm^2^ area for a CZT detector was observed to vary over a wide range of 0.2–20 × 10^–3^ cm^2^/V with an average (µτ)_e_ of 1.1 × 10^–3^ cm^2^/V^22^. This wide spatial variation of (µτ)_e_ eventually broadens the photo peak and degrades the detector’s energy resolution.

Thus, it is critically important to attain very high spatial charge-transport homogeneity to achieve a high throughput of high-quality detectors and lower the cost of production. At present CZT material does not perceptibly satisfy this criterion due to the presence of high concentrations of Te inclusions and sub-grain boundary networks. Another alternative detector material, TlBr, benefits from the absence of precipitates/inclusions in the bulk crystal; however, it suffers from a persistent contact problem, which affects the long-term stability of the detectors^23^. As a result, a frequent switching polarity of bias voltage is needed to enhance the detector lifetime of TlBr^24^.

Defect engineering has been a critical step in controlling the transport characteristics of electronic devices, and the ability to tune, reduce or annihilate material defects is essential to enable next-generation electronic devices. Currently the issues pertaining to the material properties of CZT are still challenging. One possible reason could be that the advancement of the material quality has slowed and may be approaching a plateau due to CZT’s inherent poor thermo-physical properties at near and below the material’s melting point. We have recently demonstrated that introducing selenium in CZT matrix can successfully mitigate the disadvantages in today's CZT material including better compositional homogeneity^25–28^ and enhanced mechanical hardness^29^. Efforts have been underway to improve the material through optimization of growth parameters for the last four years. Previously we obtained an energy resolutions in the range of 0.9–1.1% at 662 keV for Frisch grid detectors with a length of ~ 1 cm^26,27^. Here we report detailed characterization of high resolution virtual Frisch grid detector fabricated from as-grown Cd_0.9_Zn_0.1_Te_0.98_Se_0.02_ ingot grown by the THM technique after optimizing the growth conditions. The detector sample was characterized by IR transmission microscopy to investigate the presence of secondary phases, X-ray topography using synchrotron light source to visualize the presence of sub-grain boundaries and their networks, and residual stress. An impurity analyses of the grown material was carried out using glow discharge mass spectroscopy (GDMS), which suggests the material can be further improved by purification of the starting material before the final growth. The detector performance was assessed for the wide range of gamma energies from ~ 31 keV to 1.33 meV.

## Results

Cd_1−x_Zn_x_Te_1−y_Se_y_ (CZTS) ingots with two-inch diameter were grown by the traveling heater method (THM) using 6 N purity Te (Alfa Aesar) as the solvent with a nominal composition of 10% (atomic) of Zn and 2% of Se (atomic).

Very high compositional homogeneity of the Cd_0.9_Zn_0.1_Te_0.98_Se_0.02_ ingots were achieved as compared to CZT^27^. More specifically, about 90% compositional uniformity along the growth length for the Cd_0.9_Zn_0.1_Te_0.98_Se_0.02_ ingot^26^ was attained, which is very high compared to CZT ingots grown by the THM technique^30^. In addition, low-temperature photoluminescence (PL) mapping reveals excellent radial compositional homogeneity for the Cd_0.9_Zn_0.1_Te_0.98_Se_0.02_ ingots grown by the THM technique^26^. All the CZTS compounds with Se composition ranging from 1.5 to 7% with 10% Zn (fixed for all compounds) were consistently found to be free from sub-grain boundary networks with the occasional presence of isolated individual sub-grain boundaries^25–28^. Boosted hardness compared to conventional CZT was also demonstrated^29^. Thus, selenium addition to the CZT matrix was shown to be an important element in defect engineering for CZT-based alloys, and dramatic improvements were achieved in reducing deleterious defects present in CZT including a significant reduction of Te inclusions^25–27^.

**Figure 1:**
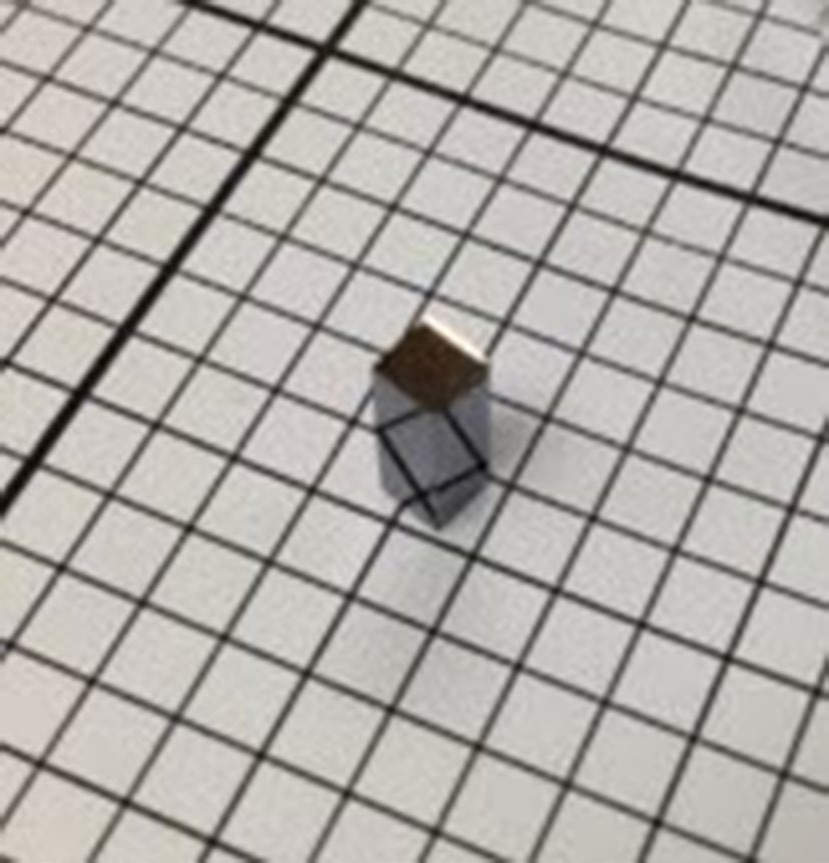
Optical photograph of the VFG detector sample with a gold contact on each end face. The sample dimensions are 3.5 × 3.5 × 9.15 mm^3^. The ruler on the same graph paper is displayed in the inset of Fig. 5b.

**Figure 2:**
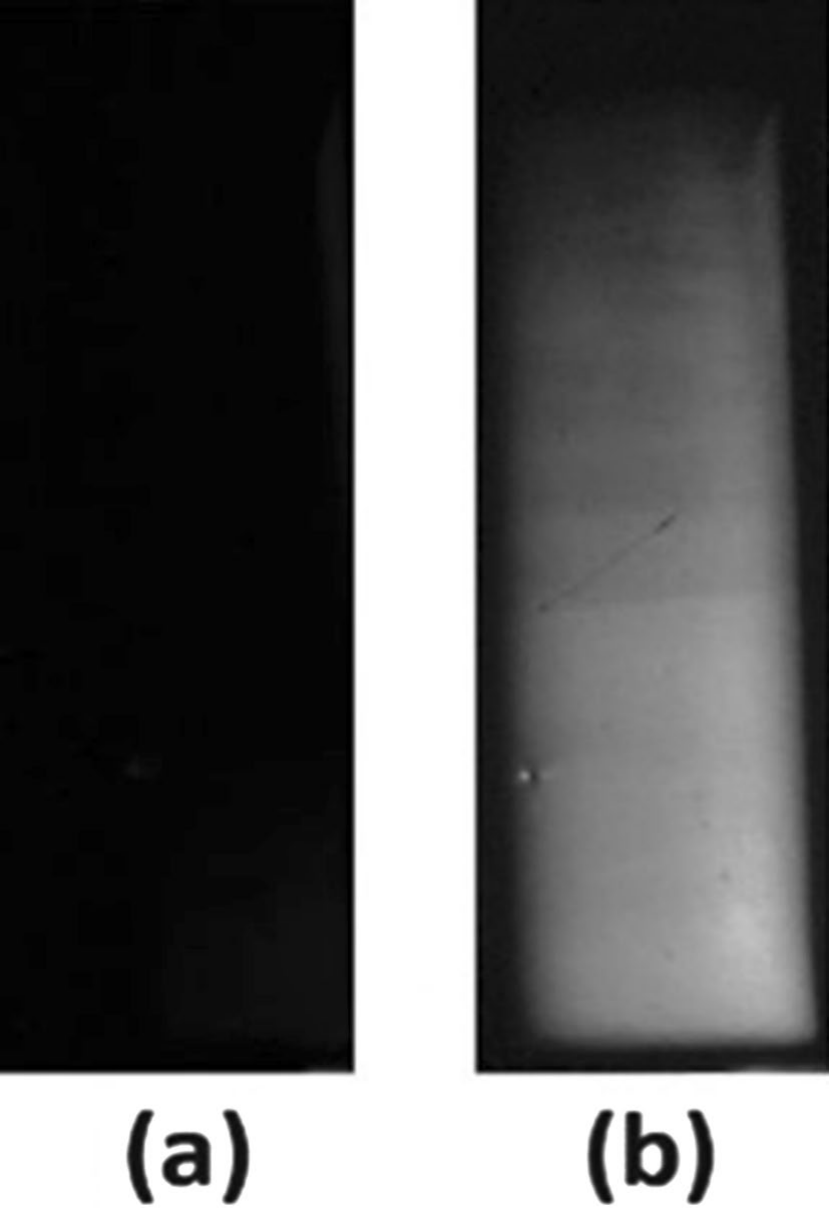
Cross polarized IR transmission images for an as-grown Cd_0.9_Zn_0.1_Te_0.98_Se_0.02_ sample: under (**a**) zero bias and (**b**) under the applied bias. Cathode is placed at the bottom surface and the anode on the top surface of the sample.

Comprehensive evaluation of the detector material fabricated in a virtual Frisch-grid (VFG) geometry from as-grown Cd_0.9_Zn_0.1_Te_0.98_Se_0.02_ ingots was performed, and the device characteristics were evaluated. Figure 1 shows an optical photograph of a typical detector sample of dimensions 3.5 × 3.5 × 9.15 mm^3^ with a gold contact on each end face. The VFG detectors are simple bar-shaped devices with high geometrical aspect ratio, which ensures single carrier (electron-transport-only devices) operation and improvement in the energy resolution^31^. Details of VFG detector fabrication are discussed elsewhere^27,31^. Prior to fabricating the detectors, the detector samples were characterized to evaluate the material defects such as Te inclusions, sub-grain boundaries and residual thermal stresses. Crossed polarized infrared (IR) transmission imaging was used for qualitative evaluation of the presence of internal stress in the samples^32^. The schematic of the experimental set up is shown in Supplementary Fig. 1. For an ideal sample, the transmission image through crossed polarizer appears dark, while stress-induced birefringence causes localized transmission of light through the sample. The presence of significant residual thermal stress is generally observed in commercial CZT samples as measured by IR transmission image under a crossed polarized condition^32^. The presence of the residual thermal stresses in CZT material, generated during the process of growth and the subsequent cooling process and even through the process of post-growth annealing^32^, is responsible for a non-uniform electric field distribution^33,34^ in the bulk of the detectors, which eventually worsens the detector performances, especially for thick devices. A uniform electric field is critically important to attain high performing radiation detectors, and thus mitigation of the thermal stress in the materials is a strict requirement. The IR transmission image under crossed polarizers for the as-grown Cd_0.9_Zn_0.1_Te_0.98_Se_0.02_ sample (shown in Fig. 1) did not show the presence of residual thermal stress as illustrated in Fig. 2. Several VFG detectors samples with different compositions of 2%, 4% and 7% Se were examined through IR transmission measurements under crossed-polarizer conditions, which reveal the absence of residual thermal stress. This elucidates that the CZTS samples are much less prone to residual thermal stress compared to conventional CZT. Figure 2b shows the same crossed-polarized IR transmission image for the sample under an applied bias of 3000 V applied on anode side. The relatively higher transmittance near the cathode side is due to the voltage dependent birefringence, which is commonly known as the Pockels effect^35^. The higher transmittance near the cathode represents a higher electric field near the cathode, which is commonly observed for CZT detectors^35^.

**Figure 3:**
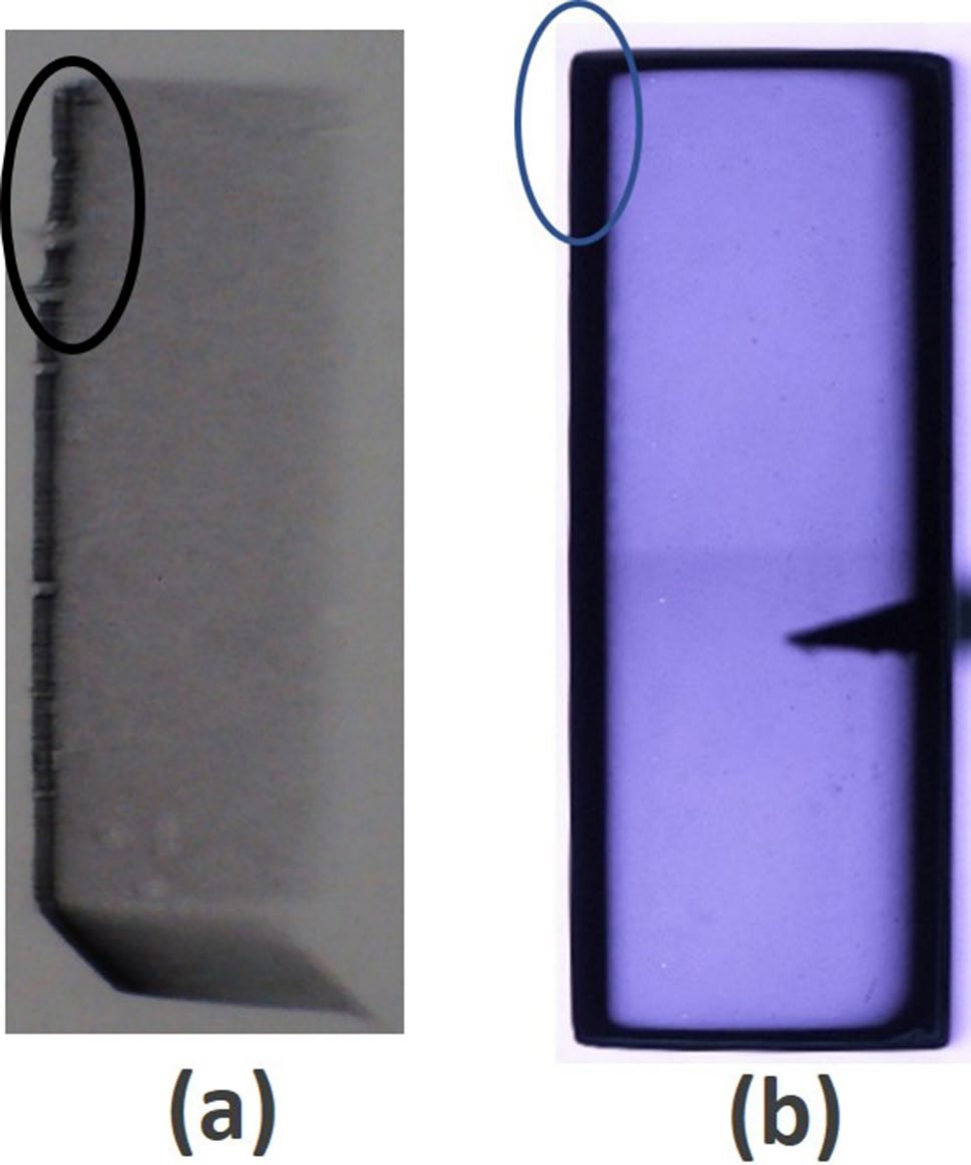
(**a**) X-ray topographic image and (**b**) IR transmission image of the as-grown Cd_0.9_Zn_0.1_Te_0.98_Se_0.02_ detector sample. Slight bending on the top left side of the sample is highlighted by the ellipse.

X-ray topography is another means to qualitatively measure thermal stress in crystalline materials. X-ray topographic images can be more sensitive to stress compared to the crossed polarized IR transmission image, as the topographic image reveals structural deformation due to stress induced lattice-distortion^18^. The topographic image deformation is more prominent near the edges of the strained samples^18^ even for defect-free samples, which produce featureless images. The X-ray topography image of the detector sample shown in Fig. 3a illustrates the slight bending on the top left side of the sample, as highlighted by the ellipse. At present it is not clear if this bending is caused by thermal-stress induced lattice deformation or by the physical deformation of the sample due to surface processing, such as polishing and bromine-methanol etching on the sides of the sample. The IR transmission image of the same sample shown in Fig. 3b also shows slight bending at the edge on the same position of the sample. It should be noted that the sample was etched in 2% bromine-methanol solution for two minutes prior to X-ray topographic measurements to minimize the likelihood of any artifact in the X-ray topographic images. The bromine-methanol etchant removes the damaged layer that is produced during polishing and handling of the sample. The enlarged version of the left top corner of the IR transmission image is shown in Supplementary Fig. 2, demonstrating the physical bending near the left top side of the sample.

Some missing parts on the left side of the X-ray topographic image (Fig. 3a) are possibly due to chipping at the edge during the lapping and polishing processes. The most striking nature of the X-ray topographic image is the featureless characteristics that reveal the absence of sub-grain boundaries and their network. This is a dramatic improvement in CZTS that has been achieved as compared to present day commercial CZT. CZT materials are commonly found to be heavily decorated with sub-grain boundary network, as often observed by X-ray topography technique^18,36^. These sub-grain boundaries impede electron transport, and the CZT community is still striving to find ways to prevent the formation of dislocations or sub-grain boundary network. The appearance of white and dark lines based on the tilt angle of adjacent sub-grains in the X-ray topographic image demonstrate the presence of sub-grain boundaries and their network. The white and dark lines are due to diverged and converged diffracted images of adjacent sub-grains respectively, depending upon the relative tilt angle of the adjacent sub-grains. The X-ray topographic image of the detector sample (Fig. 3a) shows the absence of sub-grain boundaries and their network. The absence of such defects ensures high spatial homogeneity of the charge-transport characteristics. The absence of a sub-grain boundary network has been observed to be very consistent over the entire range of selenium concentration from 1.5% (atomic) to 7.0% (atomic). However, some occasional presence of isolated individual sub-grain boundary was observed in some samples^25–28^. We conclude that selenium acts as an effective solid solution hardening agent to arrest the formation of sub-grain boundary networks in the CZTS matrix during growth and subsequent cooling processes. It is well known that certain dopants can effectively diminish the formation of sub-grain boundaries^37^, which agrees well with our present findings.

**Figure 4:**
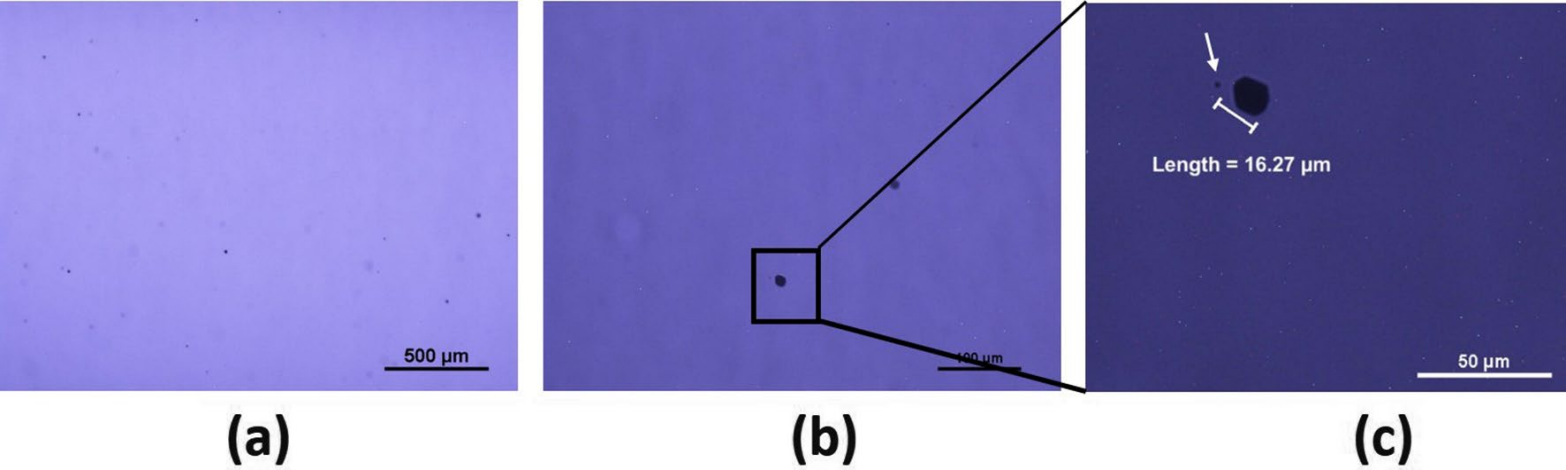
High magnification IR transmission images with higher magnifications from left to right. Length of the Te-inclusion indicated in (**c**) is 16.27 µm. The scale bars indicated on bottom right corners correspond to 500 µm, 100 µm and 50 µm for figure (**a**–**c**) respectively.

The infrared transmission imaging technique was used to investigate the presence of secondary phases, e.g., Te inclusions. Since the Te inclusions are opaque and CZTS is transparent in the infrared wavelength region, Te inclusions appear as dark spots with various shapes in the IR transmission images. The IR transmission image of as-grown Cd_0.9_Zn_0.1_Te_0.98_Se_0.02_ detector sample (shown in Fig. 1) was investigated, and very few Te inclusions were observed as shown in Fig. 3b, emphasizing dramatic improvement in material quality by reducing the Te-rich secondary phases. The dark triangular patch on the right side of the IR transmission image (Fig. 3b) is due to the piece of paper placed near the bottom edge of the sample to avoid any scratches to the bottom surface of the sample. The schematic of the sample mount on the glass plate of the microscope is shown in Supplementary Fig. 3. We have successfully reduced Te inclusions for the CZTS materials over the range of selenium concentration from 2% (atomic) to 7.0% (atomic). We note that high concentration of Te inclusions were observed for CZTS with less than 1.5% (atomic) of Se^28^. The reason for the drastic reduction of Te inclusions for the compositions of Se in the range of 2.0–7.0% (atomic) is not clear. A plausible explanation is a reduced bulging of the retrograde solidus line near stoichiometry due to the incorporation of Se^38^ in CZT, resulting in reducing the concentration of secondary phases by an order of magnitude compared to conventional CZT. The concentration reduction of Te inclusions were also observed in both Bridgman- and THM-grown CdTeSe ingots^38,39^, which elucidates the role of selenium in alleviating the concentration of Te inclusions. High magnification IR transmission microscopy was used to further investigate Te inclusions in CZTS matrix. Figure 4a–c show high magnification IR transmission images with successively increasing magnification. Figure 4c shows a hexagonal Te inclusion of size ~ 16 µm. Very few Te inclusions were observed, illustrated in the high magnification IR transmission images, as compared to conventional CZT. We observed the presence of Te inclusions with sizes in the range of 10–20 µm with very low concentrations, however, very small size (about 1 µm diameter) Te inclusions were also registered as depicted at the tip of the arrow in Fig. 4c. Inclusions of size smaller than 1 µm could not be detected due to the limitation of IR wavelength used for this investigation. On the other hand, CZTS with 1.5% (atomic) Se revealed the presence of high concentrations of Te inclusions (see Supplementary Fig. 4 for comparison), which is similar to that of conventional CZT. The presence of a very low concentration of secondary phases in as-grown Cd_0.9_Zn_0.1_Te_0.98_Se_0.02_ is very compelling. Since the secondary phases severely hinder the charge transport in practical devices, the presence of a lesser number of secondary phases ensures higher spatial homogeneity of charge-transport characteristics and thus has the potential to increase the yield of high-quality detectors. In this study, the addition of selenium in CZT matrix was found to be very effective in causing a drastic reduction of Te-rich secondary phases and annihilating the sub-grain boundary network, offering a promising approach to produce high-quality CZTS quaternary materials. Selenium thus plays a critical role in the CZT matrix for defect engineering to resolve the long-standing issues associated with CZT.

**Figure 5:**
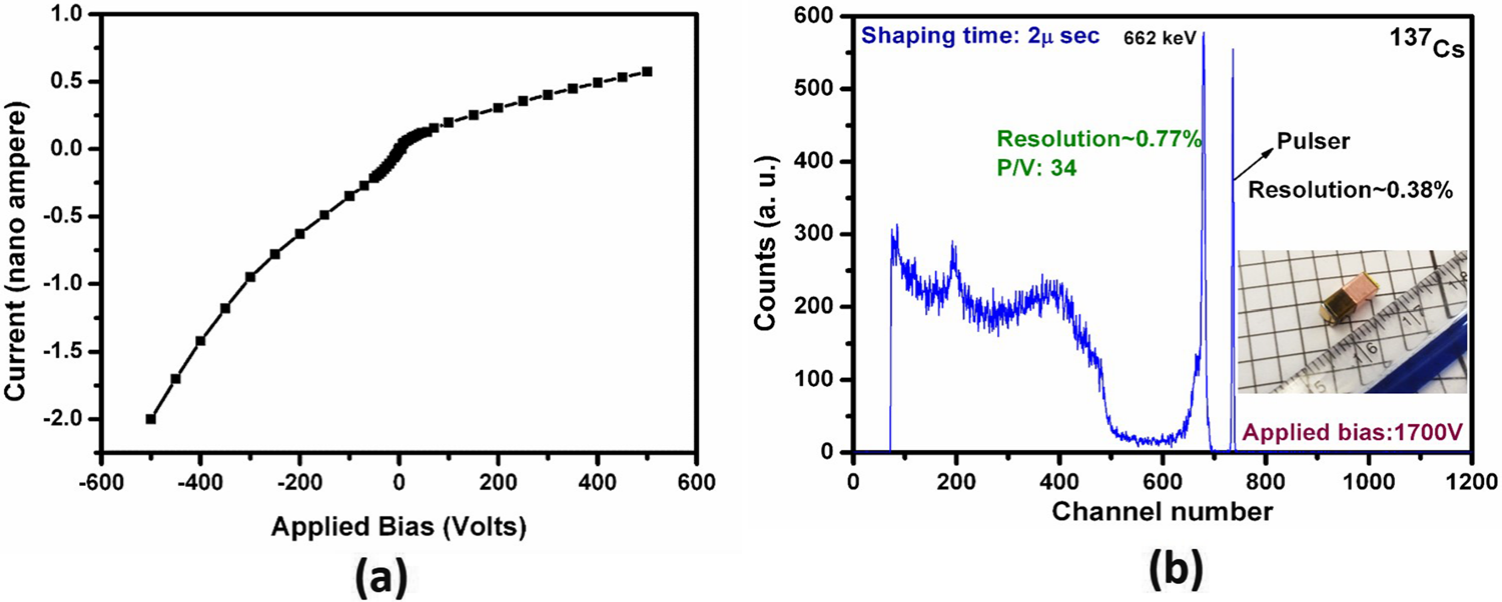
(**a**) Dark I–V characteristics of the FG detector at room temperature and (**b**) pulse height spectrum of ^137^Cs source of the Frisch-grid detector (shown in the inset) fabricated from as-grown Cd_0.9_Zn_0.1_Te_0.98_Se_0.02_ THM-grown ingot. Here, P/V is commonly known as Peak-to-Valley. It is the ratio of the peak count at 662 keV to the minimum count in the valley region (in the range of ~ 480–600 channel number. Detector dimensions: 3.5 × 3.5 × 9.15 mm^3^.

The dark current for the CZTS detectors commonly dominates the electronic noise. Lower dark current enhances the signal-to-noise ratio, which elevates detector performance. To evaluate the magnitude of the dark current, current–voltage (I–V) characteristics of the gold-contacted sample were measured prior to fabrication into the VFG detector design. A characteristic I–V plot for the detector sample over the voltage range ± 500 V is shown in Fig. 5a. The asymmetric nature of the I–V plot is commonly observed for CdTe/CdZnTe based samples and depends on surface preparation conditions, contact resistance, electrode material and Schottky barrier height; it also depends on the presence of Cd- or Te-rich surfaces left after chemo-mechanical or other surface processing^40,41^. A dark current of less than 1 nA was obtained at 500 V, which is acceptable for detector operation. The attained resistivity was ~ 1.5 × 10^10^ Ω-cm, as calculated from the slope of the I–V curve in the 0–1 V range (see Supplementary Fig. 5). Both the resistivity and the dark current satisfy the requirement for high-quality detectors with low electronic noise. The mobility-lifetime product for electrons [(µτ)_e_] for the as-grown Cd_0.9_Zn_0.1_Te_0.98_Se_0.02_ samples was in the range of 4.0–6.5 × 10^–3^ cm^2^/V^26,27^.

**Figure 6:**
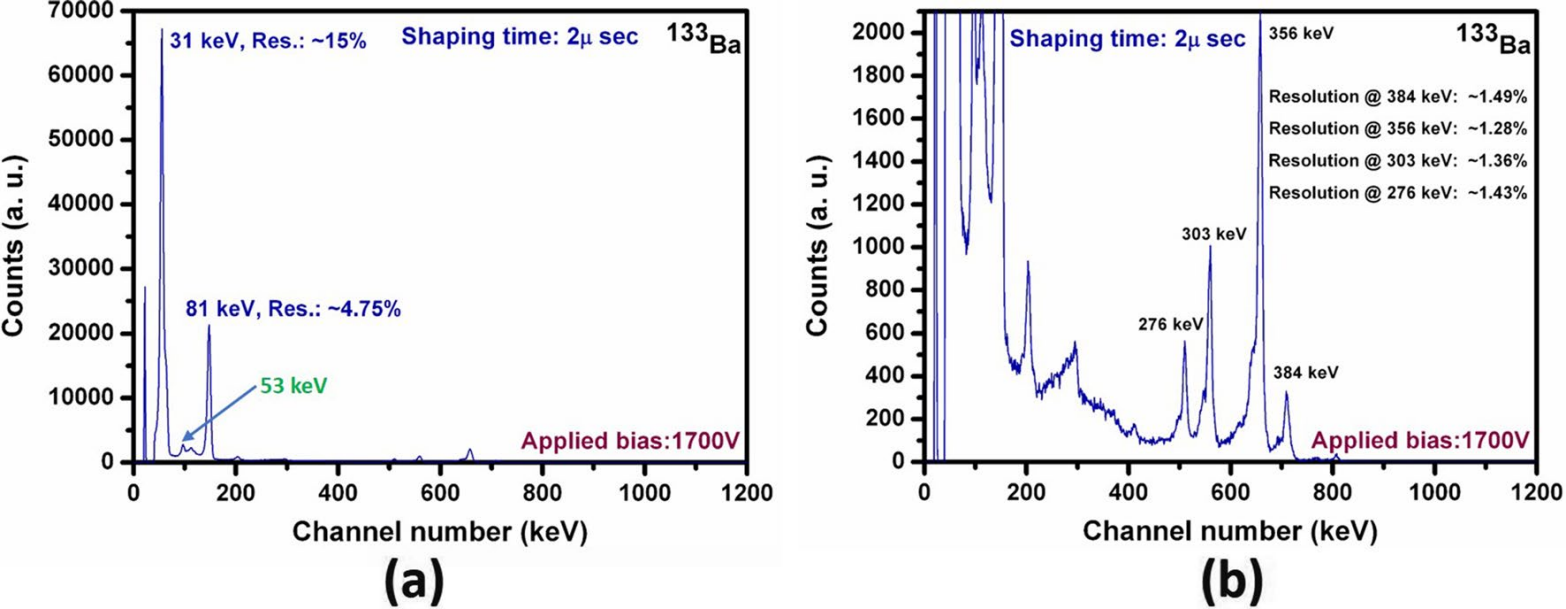
(**a**) Pulse height spectrum of ^133^Ba source of the Frisch-grid detector fabricated from as-grown Cd_0.9_Zn_0.1_Te_0.98_Se_0.02_ THM-grown ingot and (**b**) magnified version of data in (**a**). Detector dimensions: 3.5 × 3.5 × 9.15 mm^3^.

Various un-collimated radioactive point sources were used to evaluate the gamma-ray spectral responses of the detectors over a wide range of gamma energies from ~ 31 keV to 1.33 meV. Excellent energy resolution at 662 keV was achieved for the as-grown VFG detector. An energy resolution of 0.77% was obtained for an optimum Frisch grid length of 4 mm and shaping time of 2 µs, as obtained at room temperature (~ 21–23 °C) using an uncollimated ^137^Cs source (Fig. 5) under an optimum applied bias of 1700 V and shaping time of 2 µs. The pulse height spectrum shown in Fig. 5 is as-measured without any additional charge-loss corrections. To the best of our knowledge, the measured resolution (before correction) is the best to date for a room temperature semiconductor detector with this active volume. The fabricated VFG detector of dimensions 3.5 × 3.5 × 9.15 mm^3^ with the optimum VFG length of 4 mm is shown in the inset of Fig. 5. Here, the shielding grid was attached to the cathode side. For all the detector measurements, the cathode face of the detector was irradiated with gamma rays. The sharp peak with a resolution of 0.38% (Fig. 5) of the electronic pulse elucidates very low electronic noise for the device, which results from the extremely low leakage current of the detector. The detector performance for the low-energy gamma region was appraised by exposing the detector with a ^133^Ba point source (un-collimated). A very well resolved spectrum with superior energy resolutions for all the gamma lines from ^133^Ba could be achieved under the same applied bias of 1700 V with the as-grown CZTS detector. The energy resolution of the major low energy gamma peak of ^133^Ba (at 81 keV) achieved was ~ 4.75% (Fig. 6a), and the corresponding value for the 356-keV line was ~ 1.28% (Fig. 6b). Point sources of ^60^Co and ^22^Na were used for performance evaluation of the detector in the high energy region (~ 1 meV and above).

**Figure 7:**
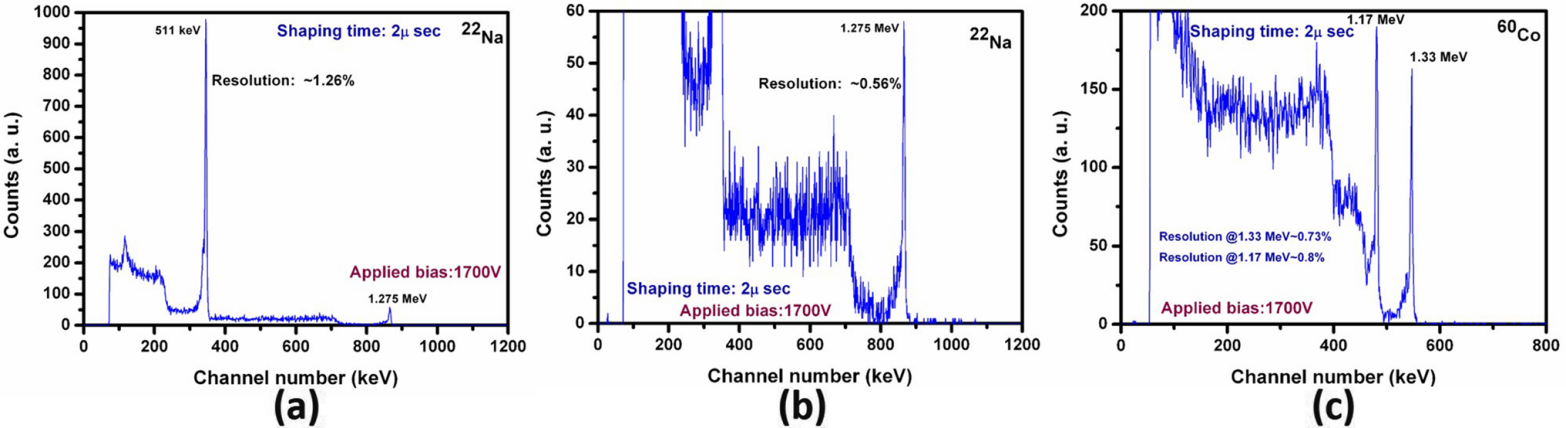
Pulse height spectrum of (**a**) ^22^Na source of the Frisch-grid detector fabricated from as-grown Cd_0.9_Zn_0.1_Te_0.98_Se_0.02_ THM-grown ingot, (**b**) magnified version of (**a**), and (**c**) ^60^Co energy spectrum. Detector dimensions: 3.5 × 3.5 × 9.15 mm^3^.

The mid energy range characteristic line at 511 keV for ^22^Na generated by the VFG detector was very well resolved with an energy resolution of ~ 1.26%, as illustrated in Fig. 7a, while the energy resolution of the high energy gamma line of ^22^Na at 1.275 meV was ~ 0.56% (Fig. 7b). The spectroscopic evaluation from a ^60^Co point source is illustrated in Fig. 7c, depicting very high-resolution peaks with energy resolutions of ~ 0.80% and ~ 0.73% for the gamma lines at energies of 1.17 meV and 1.33 meV. All the spectra presented here were as-measured (i.e., without any further charge-loss corrections). The detector measurements were performed at room temperature (~ 21–23 °C) on as-grown CZTS samples without post-growth annealing.

The overall detector performance is excellent. We are routinely producing detectors of similar dimensions fabricated from as-grown CZTS ingots with an energy resolution in the range of ~ 0.8–1.2% at 662 keV. This clearly reveals the high material quality for the new quaternary compound CZTS.

It should be noted that the only reported similar energy resolution for CZT-based VFG detectors with comparable dimensions (3 × 3 × 9 mm^3^) was 0.78% at 662 keV. A ~ 0.8% energy resolution was achieved for a detector with dimensions of ~ 4 × 4 × 11 mm^3^ for a CZT detector with a relatively high (µτ)_e_ value of > 10^–2^ cm^2^/V^42,43^. Such high (µτ)_e_ values of > 10^–2^ cm^2^/V are common in present-day commercial CZT, although the energy resolution typically achieved is > 1% at 662 keV for VFG detector designs. In the present case for the quaternary compound CZTS, the unprecedented energy resolution of 0.77% at 662 keV was measured with material having a lower (µτ)_e_ value of 4–6.5 × 10^–3^ cm^2^/V. The dramatic improvement of detector performance with reasonably moderate (µτ)_e_ value in CZTS is believed to be due to the enhanced spatial charge-transport uniformity from the presence of a very low concentration of Te inclusions and the absence of sub-grain boundaries and their network in the matrix. Considering that CZT materials are often heavily decorated with sub-grain boundaries and high concentrations of Te inclusions, the theoretical (µτ)_e_ value for CZTS is expected to be higher than that for CZT, thanks to the lower density of trapping centers connected to sub-grain boundary networks and Te inclusions. To investigate the cause for the relatively lower (µτ)_e_ value for CZTS, the purity of the material was carefully analyzed using the Glow Discharge Mass Spectroscopy (GDMS) technique. The GDMS measurements were performed at EAG Laboratories for the as-grown Cd_0.9_Zn_0.1_Te_0.98_Se_0.02_ samples grown by the THM technique. To our surprise, the content of detrimental impurities such as Cr, Fe, Ni, Cu, and others are high compared to commercial CZT. These impurities are known to introduce deep trapping centers, which may be responsible for the lower (µτ)_e_ value and for limiting the detector performance. The concentrations of Cr, Fe, Ni, and Cu present in THM-grown Cd_0.9_Zn_0.1_Te_0.98_Se_0.02_ ingot is < 20 ppba, 42 ppba, < 4 ppba and 22 ppba (parts per billion atomic) respectively. These impurity levels present in CZTS are much higher compared to those measured in commercial detector-grade CZT. In fact, the above impurities were not detectable in commercial CZT as analyzed by GDMS technique except for Fe (at 22 ppba)^44^. The incorporation of these impurities is likely from the CdSe precursor, although the stated purity of raw materials is 6 N. It is very unlikely that the source of impurities is from the CZT precursor, as CZT is consistently produced with high purity by the supplier and distributed widely to multiple institutions for commercial uses. Moreover, the impurity concentrations in the CZT raw material are much lower than those observed in CZTS. Despite the presence of such high concentrations of detrimental extrinsic impurities in the THM-grown CZTS, achieving such high energy resolution (0.77–1.2%) in CZTS detectors is very compelling from the standpoint of further improvement of the detector performance by material purification. We envisage that purified CZTS detectors offer the potential to approach high-purity, germanium-like energy resolutions following the charge-loss correction schemes typically used for demonstrating the best CZT detector performances.

In this report we demonstrated very high-energy-resolution spectroscopic radiation detectors fabricated from an as-grown CdZnTeSe ingot. The as-measured energy resolution of the virtual Frisch-grid detector at 662 keV was well below 1%. The improved material properties of CZTS after adding Se in CZT matrix are very intriguing, and they may resolve some of the long-standing material issues pertaining to CZT. The advantages of the addition of selenium in the CZT matrix was found to be four-fold. Selenium plays a critical role in arresting the formation of sub-grain boundary networks, increasing the overall hardness of the quaternary, steering the zinc segregation towards better compositional homogeneity, and drastically reducing the concentration of Te inclusions. No additional post-growth annealing process was necessary for CZTS. The post-growth annealing process is being routinely used in industry for CZT, and it is a major obstacle for realizing large detectors of non-standard dimensions due to diffusion concerns, despite intense research to optimize the annealing parameters for different sizes of detector blanks. The absence of sub-grain boundary networks and substantially reduced Te inclusions contribute to the higher spatial homogeneity of CZTS charge-transport properties, hence increasing the yield of high-quality detectors. Better compositional homogeneity also increases the overall yield of detector grade materials. The presence of high concentrations of deep-level extrinsic impurities, which are detrimental and largely responsible for the degradation of detector performance in the as-grown CZTS ingots indicates that the detector performance can be further improved by purification of the starting materials. Thus, the purified CZTS has enormous potential to approach high purity, Ge-like detector performance at ambient temperature operation. Considering the overall superior material properties and homogeneity, CZTS shows potential to supersede CZT in the future.

## Methods

The starting materials used were 99.9999% (6 N) pure Cd_0.9_Zn_0.1_Te procured from 5 N Plus, Inc., and 99.9999% pure CdSe procured from Azelis, Inc. The compound Cd_0.9_Zn_0.1_Te_0.98_Se_0.02_ was first synthesized and used as the starting material for growth. The CZTS crystals were grown by the THM technique. The THM technique has traditionally been used by industry to grow CdTe and CZT ingots. In the THM technique, the ingots are grown from a Te-rich solution zone. There are several advantages of THM technique as compared to a melt-growth technique. For growth from a Te-rich solution, the crystals can be grown at temperatures well below the melting point of the material. The lower temperature for growth offers less defects and less incorporation of extrinsic impurity migration from the crucible, plus better compositional homogeneity^44^. In addition, THM itself is a self-purification technique because of the impurity gettering capability of Te^45,46^. Thus, the ingots grown by the THM technique are purer and contain fewer defects with better compositional homogeneity as compared to the traditional melt-grown CZT-based ingots. The THM technique thus evolved as a major mode of growth for the community dealing with CdTe and related compounds, especially for industrial production. The detectors used in this present work were fabricated from as-grown Cd_0.9_Zn_0.1_Te_0.98_Se_0.02_ ingot grown by the THM technique. Similar generic growth parameters were used earlier to grow as-grown detector-grade CZT ingots^47^, were employed in this present work for CZTS. 6 N purity tellurium was used as the solvent (from Alfa Aesar) and 6 N purity indium as the dopant procured (also from Alfa Aesar) for growth of both CZT and CZTS.

IR images were taken under a cross-polarizer geometry for the transmission measurements. Images with and without applied bias were obtained using a high-resolution Guppy F-046 CCD camera with a resolution of 782 × 582 pixels from Allied Vision Technologies. The experimental procedures for microscopic imaging, X-ray topography and detector evaluation are detailed elswhere^25–28^.

## Supplementary Information

Supplementary Figures.
