# Supplementary material for: Impact of selenium addition to the cadmium-zinc-telluride matrix for producing high energy resolution X-and gamma-ray detectors
Source: Sci Rep. 2021 May 14;11:10338. doi: 10.1038/s41598-021-89795-z (PMC8121847; doi:10.1038/s41598-021-89795-z)
Supplement: Supplementary file 1 — Supplementary Figures. [file 41598_2021_89795_MOESM1_ESM.pdf]

Supplementary Information

Impact of selenium addition to the CdZnTe matrix for producing high energy-resolution gamma-ray detectors

Utpal N. Roy, Giuseppe S. Camarda, Yonggang Cui, Ge Yang and Ralph B. James

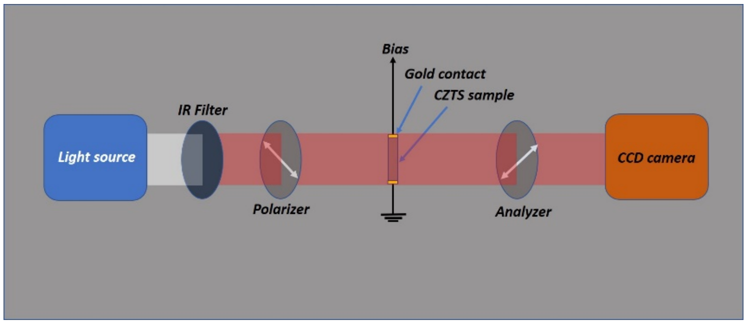

Supplementary figure 1. Experimental set up for IR transmission imaging for a crossed-polarizer configuration for measuring the Pockels effect.

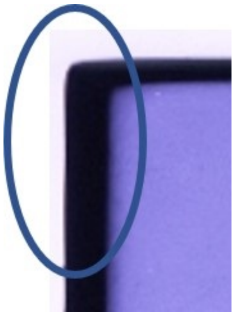

Supplementary figure 2. Enlarged IR transmission image of the detector.

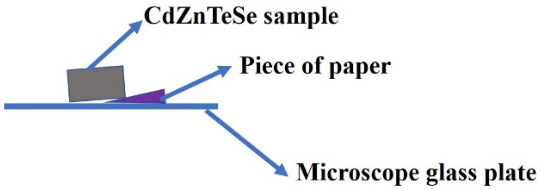

Supplementary figure 3. Schematic of the sample mounting on the glass plate of the microscope.

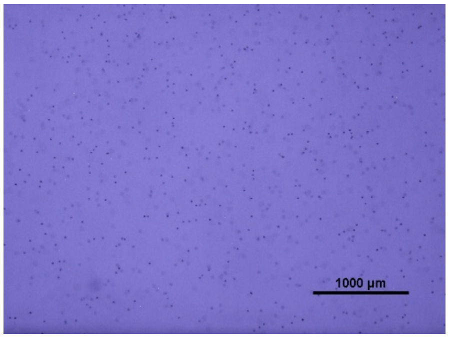

Supplementary figure 4. IR transmission microscopic image of CZTS sample with 1.5% Se concentration.

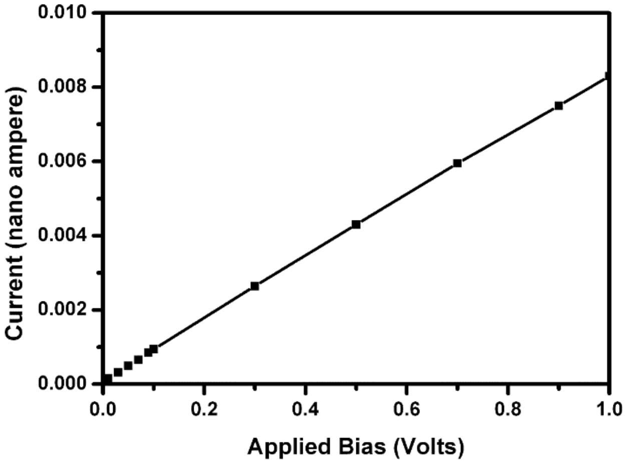

Supplementary figure 5. Current-voltage characteristics of the detector at room temperature for 0.0-1.0 V range.
